# FH-Net: Frequency-domain and hierarchical dual enhancement for few-shot surface defect segmentation

**DOI:** 10.1371/journal.pone.0358329

**Published:** 2026-09-16

**Authors:** Lili Fan, Zhanglin Yang, Fan Guo, Ping Fang

**Affiliations:** School of Mechanical Manufacturing and Automation, Chuzhou Polytechnic, Chuzhou, China; University of Electronic Science and Technology of China, CHINA

## Abstract

In real-world industrial manufacturing, surface defect segmentation is severely challenged by the extreme scarcity of annotated defect samples, which renders exhaustively supervised approaches impractical. Although meta-learning based few-shot segmentation paradigms have made initial progress, existing methods still suffer from two prominent limitations. First, the limited number of support set samples leads to insufficient feature diversity, rendering the model prone to overfitting during training. Second, feature extraction restricted to a single hierarchical level and lacking contextual guidance constrains the representational capacity for complex and multi-scale defects. To address these issues, a novel few-shot surface defect segmentation method termed FH-Net is proposed, which aims to achieve high-precision segmentation of previously unseen categories using only a limited number of annotated samples. Specifically, a Semantic-Preserving Frequency Perturbation (SPFP) module is designed, which enriches support set feature diversity through foreground- and global-level amplitude perturbations in the Fourier domain, thereby mitigating the risk of overfitting under limited supervision. Furthermore, a Hierarchical Context Aggregation Module (HCAM) is constructed, which strengthens multi-scale defect representation via parallel feature extraction branches coupled with an attention-driven adaptive weighting fusion mechanism. Extensive experiments on FSSD-12 and Surface Defects-4i demonstrate that FH-Net achieves competitive segmentation performance compared with representative recent methods under both the 1-shot and 5-shot settings. Ablation studies further confirm the contribution of each core module to the overall performance improvement.

## 1. Introduction

In the domain of industrial manufacturing, surface defect segmentation has emerged as an indispensable component of the quality control workflow [1–3]. For metallic surfaces, common defects such as inclusions, scratches, and indentations can significantly compromise the service performance of the material [4,5]. For instance, crack defects on the surface of a metallic blade introduce stress concentration points. Once crack propagation exceeds a critical threshold, catastrophic failures such as blade fracture may ensue, potentially leading to severe safety incidents. Consequently, achieving precise surface defect detection holds paramount engineering significance [6–8].

**Fig 1:**
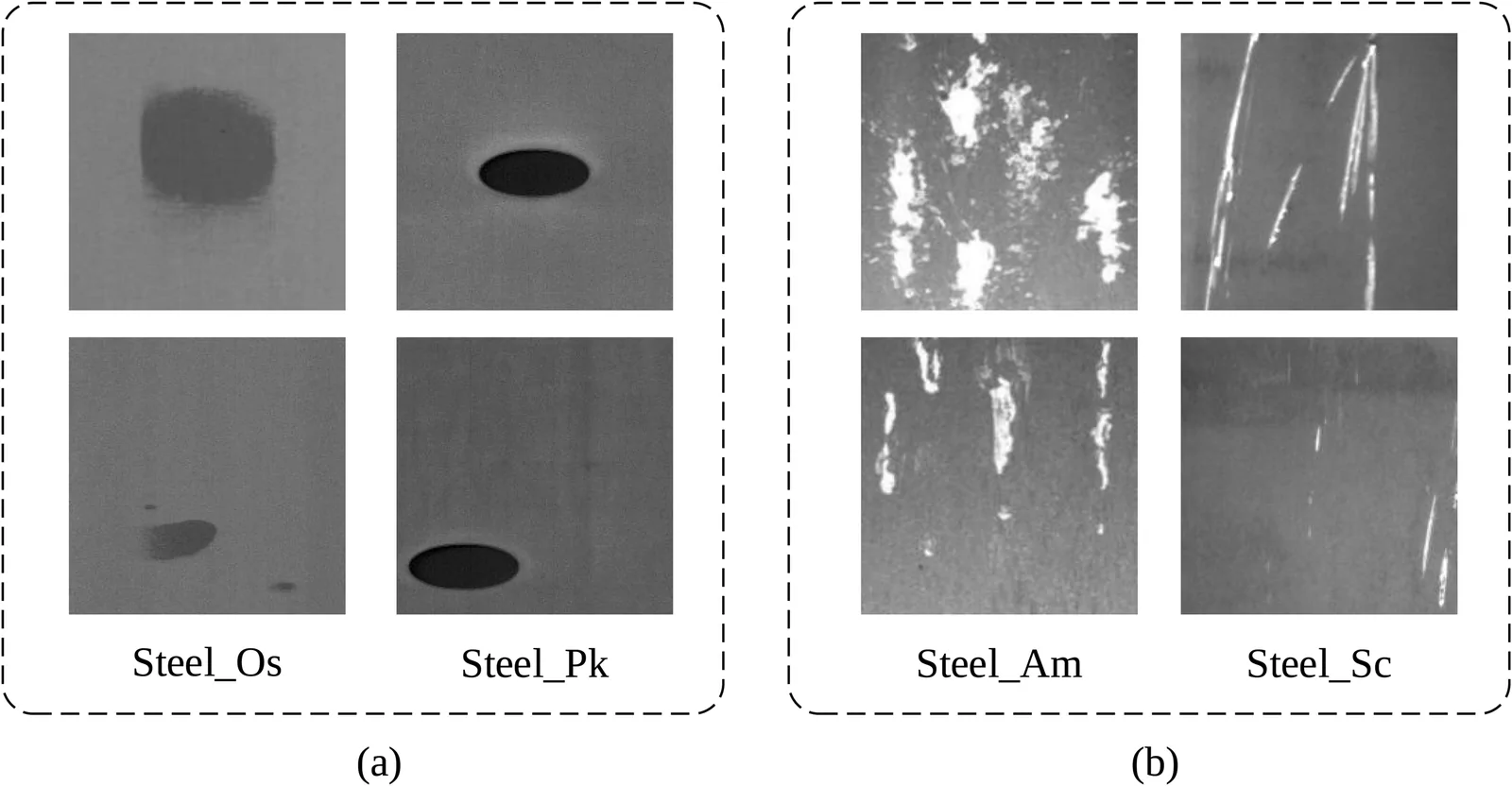
Challenges in industrial defect segmentation. (a) High inter-class feature similarity. (b) Complex defect morphologies and significant scale variation.

However, as shown in Fig 1, the intrinsic characteristics of surface defects, including pronounced intra-class scale variation, complex morphological structures, high inter-class similarity, and low contrast against textured backgrounds, pose substantial challenges to the segmentation task. Early industrial inspection relied predominantly on manual visual examination. Although this approach offers a certain degree of flexibility, it suffers from notable limitations such as strong subjectivity, low throughput efficiency, and persistently high long-term labor costs, rendering it inadequate for the demands of modern automated production. The introduction of conventional machine vision techniques has supplanted manual inspection to a certain extent and yielded moderate success. Representative technical approaches include template matching, threshold-based segmentation, and morphological processing. Nevertheless, these methods are heavily dependent on hand-crafted features and manually tuned parameters. Consequently, they exhibit limited robustness against environmental perturbations such as illumination variation and poor generalization adaptability across diverse production lines.

In recent years, the rapid advancement of deep learning has established it as a mainstream and highly effective paradigm in the domain of industrial surface defect detection [9]. Compared with conventional machine vision techniques, deep neural networks are capable of autonomously learning hierarchical feature representations, thereby exhibiting greater adaptability to complex backgrounds and heterogeneous defect morphologies. CNN-based detectors, exemplified by SSD [10] and Faster R-CNN [11], pioneered the simultaneous recognition of defect categories and coarse localization of defect regions. Subsequently, anchor-based architectures, represented by the YOLO series [12], have achieved significant improvements in inference efficiency. In contrast to bounding box regression techniques, semantic segmentation methods enable pixel-level precise delineation of defect regions, thereby capturing finer morphological details and substantially improving localization accuracy. Among them, CNN-based architectures, such as FCN [13], U-Net [14], PSPNet [15], and DeepLabv3 [16], excel at capturing local contextual variations. Meanwhile, Transformer-based frameworks, exemplified by SETR [17] and SegFormer [18], leverage the self-attention mechanism to model long-range global dependencies with a significantly broader receptive field. Driven by these advances, models tailored specifically for steel surface inspection have been successively introduced, including LGGFormer [19], TSEDNet [20], which have attained leading performance on various detection and segmentation benchmarks. However, the aforementioned methods generally rely on large-scale labeled defect datasets. In real industrial scenarios, defect instances are typically scarce and annotation is cost-prohibitive, thereby impeding the assurance of high-precision segmentation, strong generalization capability, and efficient performance. Against this backdrop, the problem of few-shot surface defect segmentation has garnered increasing attention within the research community [21].

Few-shot segmentation aims to achieve accurate delineation of novel defect categories using only a limited number of annotated support examples [22]. The prevailing approaches predominantly adopt a meta-learning paradigm, wherein a two-branch architecture consisting of a support branch and a query branch with shared feature extraction weights is employed to perform segmentation of the query image conditioned on the guidance from one or a few support samples [23]. The principal technical challenge of this task resides in effectively leveraging the information contained within the support set. Representative methods, such as CANet [24] and CPANet [25], extract prototype features from support masks to guide the query branch, thereby enhancing the model’s discriminative capacity for defects. In recent studies, competitive segmentation accuracy has been achieved using only a single support image. Nonetheless, existing methods still suffer from two notable limitations. First, owing to the extreme scarcity of support samples, the extracted prototype vectors often lack sufficient diversity and representational richness. This paucity of information renders the model prone to overfitting during the training phase, ultimately compromising its generalization capability to novel defect categories and impairing overall robustness. Second, existing approaches frequently confine feature extraction to a single scale or semantic level. Such a restriction hinders the model’s capacity to represent fine-grained spatial details when segmenting defects that exhibit drastic scale variation and irregular morphologies. The aforementioned challenges give rise to two pivotal questions:

(1) Feature enhancement under extreme scarcity: Given only a minimal number of support samples, how can the diversity of extracted prototype features be amplified to mitigate overfitting and strengthen model robustness within the meta-learning process?
(2) Multi-granularity context modeling: How can discriminative cues be extracted across multiple scales and semantic levels and subsequently fused in an effective manner, thereby augmenting the feature representational capacity of the model for fine-grained industrial defect segmentation?

To address the aforementioned limitations, a novel meta-learning based few-shot surface defect segmentation framework termed FH-Net is proposed. This method integrates a foreground frequency perturbation mechanism with a hierarchical feature aggregation strategy, aiming to enrich prototype information and amplify representational capacity, thereby achieving high-fidelity segmentation for industrial surfaces. The primary contributions of this paper are summarized as follows:

(1) A Semantic-Preserving Frequency Perturbation (SPFP) module is proposed. By performing dual-level amplitude perturbations in the frequency domain and employing learnable weighted fusion, the module enhances feature diversity while preserving semantics, thereby improving generalization and mitigating overfitting under few-shot settings.
(2) A Hierarchical Context Aggregation Module (HCAM) is introduced. It captures local, scale-aware, and global contexts via three parallel branches and adaptively fuses multi-granularity features, strengthening multi-scale interactions and reducing fine-grained information loss for improved defect segmentation.
(3) Extensive experiments conducted on two industrial few-shot surface defect segmentation benchmarks, FSSD-12 and Surface Defects-4i, demonstrate that FH-Net achieves competitive performance across multiple evaluation metrics under both the 1-shot and 5-shot settings. Ablation studies further substantiate the effectiveness and contributions of each core component within the proposed method.

The remainder of this paper is organized as follows. Section 2 reviews related work. Section 3 elaborates on the principles and key technical components of the proposed method. Section 4 presents the experimental setup and analyzes the results. Section 5 concludes the paper and outlines directions for future research.

## 2. Related works

### 2.1. Surface defect segmentation

Pixel-level segmentation of metallic surface defects has demonstrated significant advantages and plays a vital role in industrial production, as it provides fine-grained information such as defect shape, size, and boundary delineation. With the continuously increasing demand for product quality in manufacturing, the precision of surface defect detection has become a core requirement in industrial automation workflows. Consequently, this research direction has attracted widespread attention from both the computer vision and industrial quality inspection communities and has emerged as a prominent research focus. Guo et al. [26] proposed a pixel-level segmentation network for metallic surfaces named SPEED. This network adopts a dual-branch architecture, wherein one branch introduces semantic priors to capture low-level detail features, while the other employs a dilated lightweight structure to extract high-level contextual information. The integration of features from both branches enhances the overall segmentation performance. Liu et al. [27] presented a pyramid feature convolutional neural network for rail surface defect segmentation. By combining a lightweight backbone with a pyramid feature extraction structure, the method achieves efficient and accurate defect delineation. Addressing the issue of information loss for small-sized defects, Liu et al. [28] introduced TAS2-Net. This network incorporates a triple context attention module and a focal context module to capture richer defect information, thereby enabling pixel-level segmentation of tiny defects. Lu et al. [29] proposed MRD-Net, which achieves real-time, end-to-end segmentation of surface defects. Sampath et al. [30] introduced the Defect-Aux-Net framework. By leveraging rich auxiliary information from related tasks, this approach mitigates the adverse effects of imbalanced sample distributions and enhances both the robustness and accuracy of CNN-based surface defect recognition. Addressing the problem of unserviced rail surface defect detection, Wang et al. [31] presented CLANet. This network utilizes RGB images to supply complementary spatial information and incorporates a multimodal attention module along with a dual-stream decoder to highlight complex defect targets and enrich high-level features, thereby enabling precise detection.

Despite the notable progress achieved by the aforementioned methods in the domain of metallic surface defect segmentation, inherent limitations persist. First, existing approaches generally rely on large-scale annotated samples for supervised learning, and their segmentation performance tends to degrade substantially when defect samples are limited. Second, owing to the pronounced morphological discrepancies of defects across different products and materials, the generalization capability of these methods is severely constrained when confronted with novel categories or cross-material scenarios.

### 2.2. Few-shot defect segmentation

Few-shot defect segmentation methods are capable of segmenting previously unseen defect categories after being trained on samples from other domains or classes, requiring only a limited number of target domain examples. Owing to their minimal sample requirements and strong domain generalization capability, few-shot defect segmentation has stimulated extensive research efforts, leading to the introduction of a series of methods tailored for industrial applications. Shaban et al. [32] first introduced the concept of one-shot learning to the task of semantic segmentation. Subsequently, Zhang et al. [33] proposed SG-One, which uses a single support image as reference to predict the segmentation mask of a query image by establishing guidance via cosine similarity, thereby yielding more accurate results. CANet [24] presented a class-agnostic few-shot segmentation architecture that employs a two-branch dense comparison module to match multi-level features between support and query images, along with an attention module for feature aggregation, thus improving segmentation precision under few-shot conditions. Zhang et al. [34] introduced PGNet, a pyramid-structured network that treats image regions at different scales as graph nodes to perform hierarchical graph reasoning. PFENet [35] combines a prior mask generation strategy with a feature enrichment module to enhance generalization and alleviate the spatial inconsistency between support and query sets. CRNet [36] performs cross-reference comparison between predicted features of support and query images to strengthen the identification of co-occurring objects, and incorporates a mask refinement module for iterative foreground optimization. SAGNN [37] is an end-to-end scale-aware graph neural network that constructs graph nodes from query features and captures cross-scale relationships through progressive message passing, thereby effectively handling defects with substantial morphological variation. Yang et al. [38] proposed PMMs, which employs an expectation-maximization algorithm to fuse rich semantic features with support images, thereby improving semantic utilization efficiency and boosting segmentation performance. SCL [39] is a few-shot segmentation architecture that integrates self-guidance and cross-guidance by aggregating primary and auxiliary support vectors to recover critical feature information and refine final predictions. Feng et al. [25] introduced CPANet, a network tailored for strip steel surface defect segmentation. It utilizes a cross-position proxy module to capture long-range dependencies among scattered defects, a support auxiliary module to strengthen feature aggregation, and a spatial compression attention mechanism to extract multi-scale context while suppressing background interference.

Nevertheless, the aforementioned methods still suffer from several limitations. Firstly, most approaches rely solely on global average pooling for prototype extraction from support features, an operation that tends to discard rich discriminative information within local regions. Moreover, the scarcity of training samples leads to insufficient feature diversity, rendering the model susceptible to overfitting. Secondly, existing methods lack elaborate designs for cross-scale feature interaction and fall short in preserving fine-grained structural information within the features. Collectively, these limitations constrain the robustness and representational capacity of prototype features, thereby impeding further performance improvements in few-shot defect segmentation.

## 3. Methods

### 3.1. Overall framework of the FH-Net

**Fig 2:**
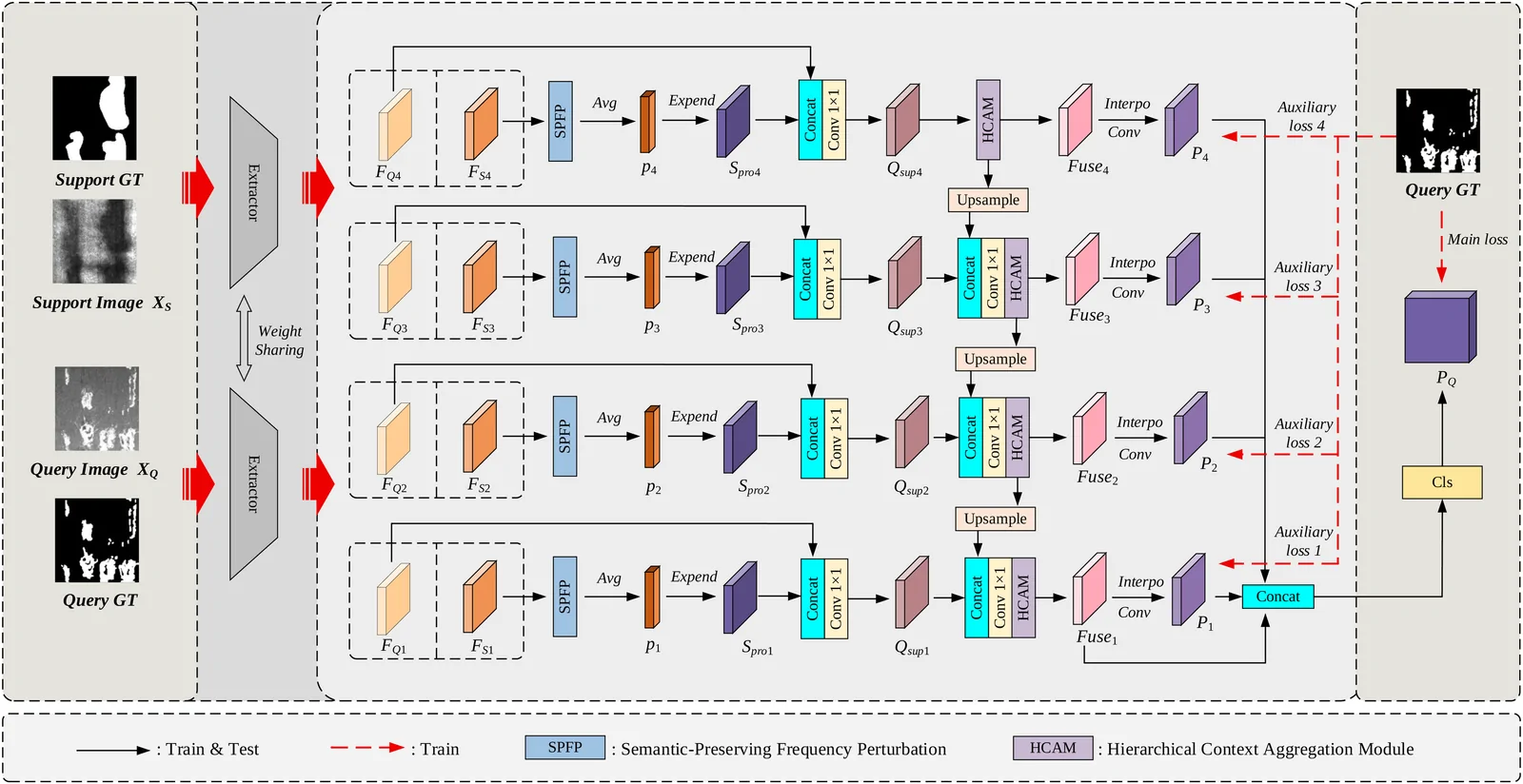
Architecture of FH-Net.

The overall architecture of the proposed model is illustrated in the Fig 2. During the training phase, the input image data consist of two categories, namely the support set and the query set. First, the support images *X*_*S*_ and the query images *X*_*Q*_ are fed into the feature extractor to obtain the support features *F*_*Si*_ and the query features *F*_*Qi*_ (where *i* = 1,2,3,4), respectively. The feature extractors for the support set and the query set share the same parameters. The support features *F*_*Si*_ are processed by the SPFP module to enhance feature representation, followed by global average pooling and feature expansion to derive the prototype features of the support set, denoted as *S*_*proi*_. The computation is formulated as follows:


$$\begin{array}{c} \left[F_{Si},F_{Qi}\right]=\mathrm{Extractor}\left(\left[X_{S},X_{Q}\right]\right),\;i=1,2,3,4 \end{array}$$ (1)

$$\begin{array}{c} p_{i}=AvgPool\left(\mathrm{SPFP}\left(F_{Si}\right)\right) \end{array}$$ (2)

$$\begin{array}{c} S_{proi}=Extend\left(p_{i}\right) \end{array}$$ (3)

where *AvgPool* denotes global average pooling, SPFP represents the Semantic-Preserving Frequency Perturbation module, and *Extend* signifies feature expansion. Subsequently, the support prototype features *S*_*proi*_ and the query features *F*_*Qi*_ are concatenated along the channel dimension. A 1×1 convolution is then applied to compress the channel dimension and fuse the two feature representations. The resulting features are further processed by the HCAM module for multi-scale contextual information aggregation, yielding the fused features denoted as *Fuse*_*i*_.The computation for this process is formulated as follows:


$$\begin{array}{c} Q_{supi}={Conv}^{k=1}\left(\mathrm{Concat}\left(S_{proi},F_{Qi}\right)\right) \end{array}$$ (4)

$$\begin{array}{c} {Fuse}_{i}=\mathrm{HCAM}\left(Q_{supi}\right) \end{array}$$ (5)

The fused features *Fuse*_*i*_ are upsampled and subsequently fused with *Q*_*sup*(*i*-1)_ in a cross-level manner. After sequentially undergoing concatenation, a 1×1 convolution, and processing by the HCAM, the features of another level, denoted as *Fuse*_*i*-1_, are obtained. This procedure is repeated to derive fused features at all four hierarchical levels. The computation is formulated as follows:


$$\begin{array}{c} {Fuse}_{i-1}=\mathrm{HCAM}\left(Conv\left(\mathrm{Concat}\left(\mathrm{UpSample}\left({Fuse}_{i}\right),Q_{sup(i-1)}\right)\right)\right) \end{array}$$ (6)

The fused features obtained from different hierarchical levels are rescaled in terms of width, height, and channel dimensions via linear interpolation and convolutional processing, yielding the features denoted as *P*_*i*_. These features *P*_*i*_ are then concatenated with *Fuse*_1_ and fed into the detection head to produce the final model output *P*_*Q*_. The computation is formulated as follows:


$$\begin{array}{c} P_{i}={Conv}^{k=3}\left(Interpo\left({Fuse}_{i}\right)\right) \end{array}$$ (7)

$$\begin{array}{c} P_{Q}=Cls\left(\mathrm{Concat}\left({Fuse}_{1},P_{i}\right)\right) \end{array}$$ (8)

where *Interpo* denotes linear interpolation, and *Cls* represents the detection head. During training, the ground truth mask of the query set is used to compute the primary loss with *P*_1_, while auxiliary losses are computed with *P*_2_, *P*_3_, and *P*_4_, respectively. The final loss of the model is obtained by summing the primary loss and all auxiliary losses.

### 3.2. Semantic-preserving frequency perturbation

**Fig 3:**
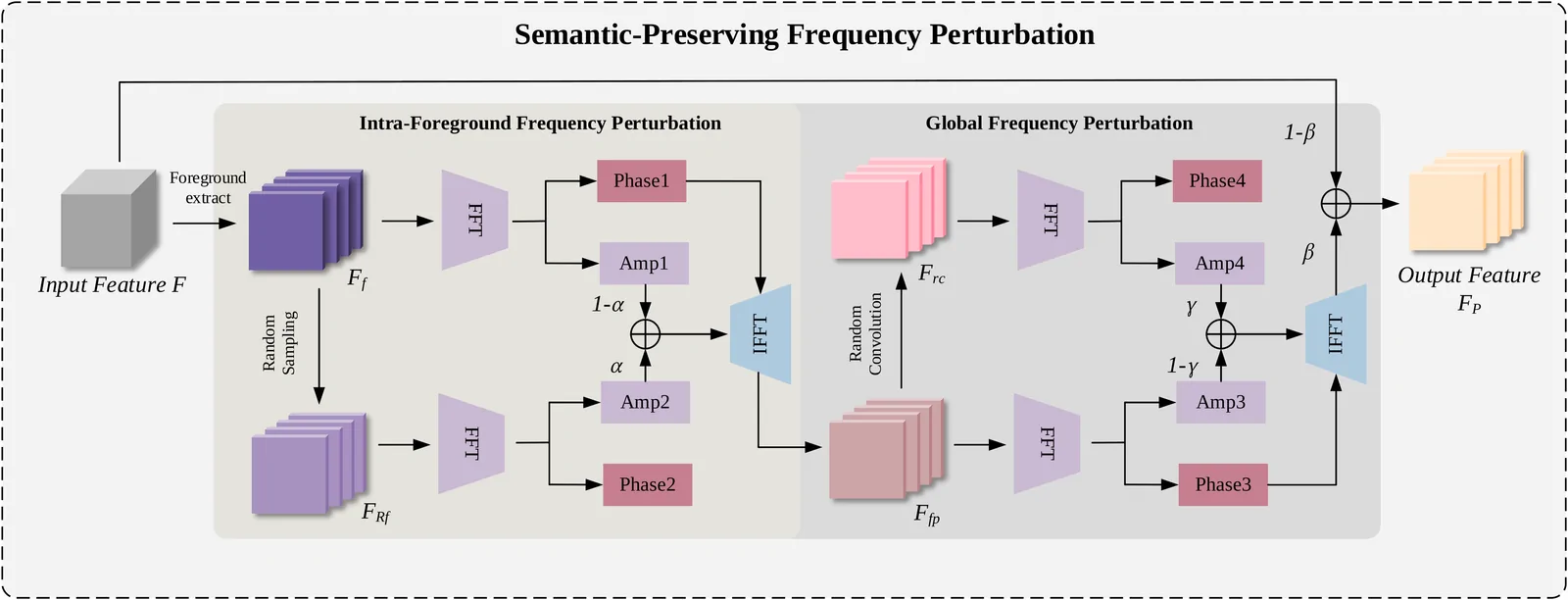
Architecture diagram of SPFP.

To address the issues of poor model learning capacity and overfitting in few-shot learning tasks, which arise from insufficient data and limited sample diversity, the SPFP module is proposed. As shown in Fig 3, this module comprises two components, namely intra-foreground frequency perturbation and global frequency perturbation. The details are presented as follows.

(1) Intra-Foreground Frequency Perturbation: First, the foreground region is extracted from the input feature *F*, yielding the feature *F*_*f*_ that contains the foreground target area to the greatest possible extent. Random region sampling is then performed within the width and height dimensions of *F*_*f*_ to obtain a local foreground patch, denoted as *F*_*rf*_. Fast Fourier Transform (FFT) is applied to both *F*_*f*_ and *F*_*rf*_ to derive their respective frequency-domain amplitude and phase components. The amplitude and phase of feature *F*_*f*_ are denoted as *Amp*_1_ and *Phase*_1_, while those of feature *F*_*rf*_ are denoted as *Amp*_2_ and *Phase*_2_. The two amplitude components *Amp*_1_ and *Amp*_2_ are aggregated via weighted fusion to obtain the fused amplitude *Amp*_*fuse*1_. The computation is formulated as follows:

$$\begin{array}{c} {Amp}_{1},{Phase}_{1}=\mathrm{FFT}\left(F_{f}\right) \end{array}$$ (9)

$$\begin{array}{c} {Amp}_{2},{Phase}_{2}=\mathrm{FFT}\left(F_{rf}\right) \end{array}$$ (10)

$$\begin{array}{c} {Amp}_{fuse1}=\alpha \times {Amp}_{2}+\left(1-\alpha \right)\times {Amp}_{1} \end{array}$$ (11)

where α is a learnable weight parameter, and FFT denotes the Fast Fourier Transform. The learnable weight adaptively adjusts the contributions of different amplitude components and controls the perturbation strength during training. After obtaining the *Amp*_*fuse*1_, the *Phase*_1_ of the foreground feature *F*_*f*_ is retained. The inverse Fast Fourier Transform (IFFT) is then applied to *Amp*_*fuse*1_ and *Phase*_1_ to reconstruct the feature *F*_*fp*_, thereby completing the intra-foreground frequency perturbation for the input feature. The computation is formulated as follows:


$$\begin{array}{c} F_{fp}=\mathrm{IFFT}\left({Amp}_{fuse1},{Phase}_{1}\right) \end{array}$$ (12)

(2) Global Frequency Perturbation: After obtaining the feature *F*_*fp*_ from the intra-foreground perturbation, a random convolution operation is applied to it, yielding the feature *F*_*rc*_. The random convolution employs randomly generated kernels that are excluded from gradient-based optimization. According to the convolution theorem, it acts as a frequency-dependent filter and generates a perturbed reference amplitude spectrum for subsequent amplitude fusion, rather than directly replacing the spatial structure of the input feature. Subsequently, FFT is performed on both *F*_*fp*_ and *F*_*rc*_ to obtain their respective frequency-domain components. The amplitude and phase of *F*_*fp*_ are denoted as *Amp*_3_ and *Phase*_3_, while those of *F*_*rc*_ are denoted as *Amp*_4_ and *Phase*_4_. The two amplitude components *Amp*_3_ and *Amp*_4_ are aggregated via weighted fusion to produce the fused amplitude *Amp*_*fuse*2_. The computation is formulated as follows:

$$\begin{array}{c} {Amp}_{3},{Phase}_{3}=\mathrm{FFT}\left(F_{fp}\right) \end{array}$$ (13)

$$\begin{array}{c} {Amp}_{4},{Phase}_{4}=\mathrm{FFT}\left(F_{rc}\right) \end{array}$$ (14)

$$\begin{array}{c} {Amp}_{fuse2}=\gamma \times {Amp}_{4}+\left(1-\gamma \right)\times {Amp}_{3} \end{array}$$ (15)

where γ is a learnable aggregation weight parameter. Subsequently, the *Phase*_3_ of the feature *F*_*fp*_, which remains unperturbed by the random convolution, is retained. The *Amp*_*fuse*2_ and *Phase*_3_ are then combined via IFFT to reconstruct the feature *F*_*gp*_, which incorporates both intra-foreground and global-level frequency perturbations. Finally, the resulting feature *F*_*gp*_ is aggregated with the original input feature *F* through a skip connection using weighted fusion, yielding the output feature *F*_*p*_. The purpose of this weighted aggregation is to preserve the semantic information of the original feature, thereby preventing excessive perturbation that could disrupt the overall feature structure. The computation is formulated as follows:


$$\begin{array}{c} F_{gp}=\mathrm{IFFT}\left({Amp}_{fuse2},{Phase}_{3}\right) \end{array}$$ (16)

$$\begin{array}{c} F_{p}=\beta \times F_{gp}+\left(1-\beta \right)\times F \end{array}$$ (17)

where β is a learnable weighting parameter. SPFP uses learnable weights to regulate the amplitude variations introduced at both perturbation stages. Random convolution generates diverse reference amplitude spectra, while retaining the original phase during reconstruction helps preserve the spatial structure of defects. The weighted skip connection further limits excessive deviation from the original feature. Consequently, SPFP increases support-feature diversity while reducing the risk of structural distortion. These effects are quantitatively verified in Section 4.3 (4). Additionally, a probability parameter prob controls whether a feature processed by intra-foreground perturbation proceeds to the global perturbation stage. The influence of this parameter on segmentation performance is evaluated in the subsequent experimental section.

### 3.3. Hierarchical context aggregation module

Industrial surface defects are often characterized by irregular morphologies, substantial scale variations, and susceptibility to interference from textured backgrounds. Feature representations based on a single receptive field or a single level of information may struggle to simultaneously preserve fine-grained defect structures, accommodate defect regions at different scales, and suppress background interference. To address these challenges, HCAM is introduced to model fine-grained structures, multi-scale context, and global semantic information through the Local, Scale-aware, and Global Branches, respectively, followed by the joint fusion of these three types of complementary features.

**Fig 4:**
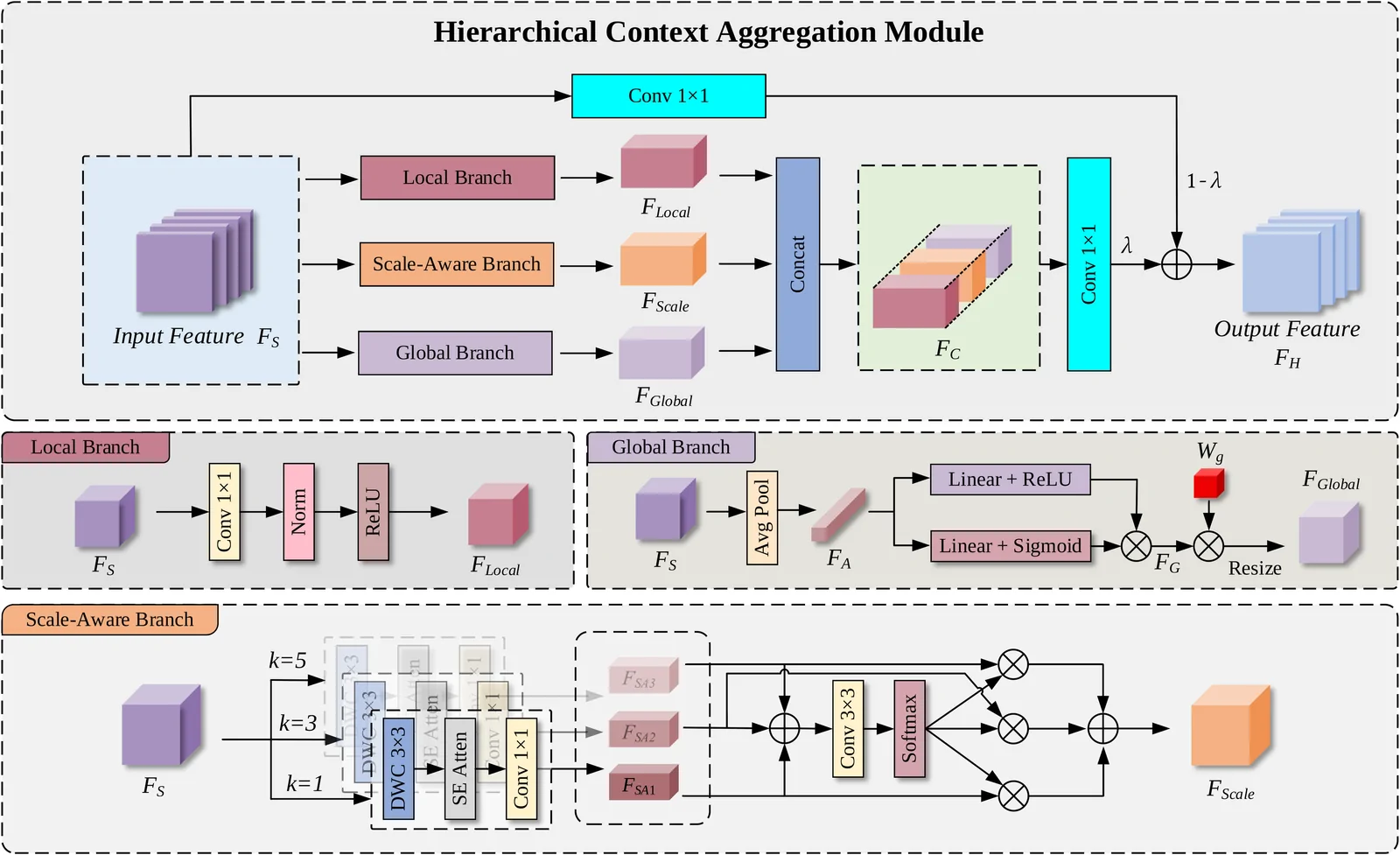
Architecture diagram of HCAM.

The overall architecture of HCAM is illustrated in Fig 4. Given an input feature $F_{S}\in R^{C\times H\times W}$, it is fed into the Local, Scale-aware, and Global Branches. These three branches extract fine-grained structural information, multi-scale contextual information, and global semantic information, producing $F_{\mathrm{Local}}$, $F_{\mathrm{Scale}}$, and $F_{\mathrm{Global}}$, respectively. All three outputs have the same spatial dimensions and number of channels as the input feature, i.e., $F_{\mathrm{Local}}$, $F_{\mathrm{Scale}}$, $F_{\mathrm{Global}}\in R^{C\times H\times W}$. This process is formulated as follows:


$$\begin{array}{c} \left[F_{\mathrm{Local}},F_{\mathrm{Scale}},F_{\mathrm{Global}}\right]=\left[f_{\mathrm{Local}}(F_{S}),f_{\mathrm{Scale}}(F_{S}),f_{\mathrm{Global}}(F_{S})\right] \end{array}$$ (18)

Subsequently, the outputs of the three branches are concatenated along the channel dimension to obtain the fused feature $F_{C}\in R^{3C\times H\times W}$, which is formulated as follows:


$$\begin{array}{c} F_{C}=\mathrm{Concat}\left(F_{\mathrm{Local}},F_{\mathrm{Scale}},F_{\mathrm{Global}}\right) \end{array}$$ (19)

To fuse the original input feature with the multi-branch contextual feature, two independent 1×1 convolutions are first applied to *F*_*S*_ and *F*_*C*_, respectively, for channel mapping, ensuring that they have the same output dimensions. The two resulting features are then fused using the learnable weight λ, producing the HCAM output feature $F_{H}\in R^{C\times H\times W}$. This process is formulated as follows:


$$\begin{array}{c} F_{H}=\left(1-\lambda \right)\times Conv\left(F_{S}\right)+\lambda \times Conv\left(F_{C}\right) \end{array}$$ (20)

where λ denotes the learnable fusion weight. The two *Conv* operations in Eq (20) represent independent 1×1 convolutional mappings applied to *F*_*S*_ and *F*_*C*_, respectively, and do not share parameters. By preserving the input feature pathway, this weighted fusion strategy incorporates multi-level contextual information while mitigating the loss of information from the original feature.

(1) Local Branch: This branch is designed to preserve the spatial correspondence and fine-grained responses of the input feature. First, a 1×1 convolution is applied at each spatial location for channel mapping and information interaction without changing the spatial resolution of the feature map. The resulting feature is then processed by normalization and *ReLU* activation to obtain the Local Branch output $F_{\mathrm{Local}}$. This process is formulated as follows:

$$\begin{array}{c} F_{\mathrm{Local}}=ReLU\left(Norm\left({Conv}^{k=1}\left(F_{S}\right)\right)\right) \end{array}$$ (21)

where *Norm* denotes the normalization operation, and *k* denotes the convolution kernel size. Since the 1×1 convolution introduces no additional spatial downsampling, this branch preserves fine-grained feature responses at each spatial location while performing channel transformation, thereby providing a local structural basis for the subsequent fusion of multi-scale and global semantic information.

(2) Scale-aware Branch: This branch is designed to extract multi-scale contextual information from different receptive fields. Given the input feature *F*_*S*_, three independent parallel pathways are constructed with convolution kernel sizes $k_{i}\in \{1,3,5\}$. In the *i*-th pathway, depthwise convolution, SE attention, and a 1×1 convolution are sequentially applied for scale-specific feature extraction, channel recalibration, and channel mapping, respectively, producing three scale-specific features *F*_*SA*1_, *F*_*SA*2_, and *F*_*SA*3_. This process is formulated as follows:

$$\begin{array}{c} \left[F_{SA1},F_{SA2},F_{SA3}\right]={Conv}^{k=1}\left(\mathrm{SE}\left({DWConv}^{k=1,3,5}\left(F_{S}\right)\right)\right) \end{array}$$ (22)

where *DWConv* denotes depthwise convolution, and $k_{i}\in \{1,3,5\}$ denotes the convolution kernel size used in the *i*-th pathway. Eq (22) represents three parallel and independently parameterized scale-specific feature extraction pathways. Convolution kernels of different sizes are used to capture contextual information under different receptive fields. In each pathway, SE attention recalibrates the channel responses of the corresponding scale-specific feature, followed by a 1×1 convolution for channel mapping and cross-channel information interaction. Compared with standard convolution, depthwise convolution reduces the parameter count and computational cost when constructing multi-scale feature extraction pathways.

Subsequently, the three scale-specific features *F*_*SA*1_, *F*_*SA*2_, and *F*_*SA*3_ are summed element-wise to obtain the aggregated feature *F*_*SA*_. A 3×3 convolution is then applied to predict three scale-specific spatial weight maps, followed by *Softmax* normalization along the scale dimension. The resulting attention weights are denoted as $\mathrm{Att}={\left\{{\mathrm{Att}}_{i}\right\}}_{i=1}^{3}$, where ${\mathrm{Att}}_{i}$ represents the spatial weight assigned to the *i*-th scale. Finally, the three scale-specific features are weighted by their corresponding attention maps and summed to obtain the output feature *F*_*Scale*_. This process is formulated as follows:


$$\begin{array}{c} F_{SA}=F_{SA1}+F_{SA2}+F_{SA3} \end{array}$$ (23)

$$\begin{array}{c} {\left\{{\mathrm{Att}}_{i}\right\}}_{i=1}^{3}=Softmax\left({Conv}_{c=3}^{k=3}\left(F_{SA}\right)\right) \end{array}$$ (24)

$$\begin{array}{c} F_{Scale}=\sum_{i=1}^{3}{\mathrm{Att}}_{i}\times F_{SAi} \end{array}$$ (25)

where ${\mathrm{Att}}_{i}$ denotes the scale-specific spatial attention map for *F*_*SA*i_. The *Softmax* operation is performed along the scale dimension, such that$\sum_{i=1}^{3}{\mathrm{Att}}_{i}\left(h,w\right)=1$ at each spatial location (*h*,*w*). This spatially varying weighting mechanism enables the branch to selectively aggregate information from different receptive fields according to the local characteristics of the defect regions.

(3) Global Branch: This branch is designed to extract global semantic information from the input feature. Given $F_{S}\in R^{C\times H\times W}$, global average pooling is first applied to compress its spatial dimensions, producing the channel descriptor $F_{A}\in R^{C}$, which is formulated as follows:

$$\begin{array}{c} F_{A}=AvgPool\left(F_{S}\right) \end{array}$$ (26)

Subsequently, *F*_*A*_ is fed into two parallel linear mappings with independent parameters. One pathway employs *ReLU* activation to generate a global semantic representation, whereas the other uses *Sigmoid* activation to produce channel-wise gating weights. The outputs of the two pathways are multiplied elementwise to obtain the gated global feature *F*_*G*_, which is formulated as follows:


$$\begin{array}{c} F_{G}=ReLU\left(Linear\left(F_{A}\right)\right)\times Sigmoid\left(Linear\left(F_{A}\right)\right) \end{array}$$ (27)

where the two *Linear* operations denote independently parameterized linear layers. The *ReLU* pathway enhances the nonlinear channel representation, whereas the *Sigmoid* pathway controls the degree of information retained in each channel.

Finally, the learnable global weight $W_{g}$ is used to modulate the response magnitude of *F*_*G*_, and the resulting channel vector is spatially expanded to *H*×*W* to obtain $F_{\mathrm{Global}}\in R^{C\times H\times W}$, which is formulated as follows:


$$\begin{array}{c} F_{\mathrm{Global}}=Resize\left(W_{g}\times F_{G}\right) \end{array}$$ (28)

where $W_{g}$ denotes the learnable global modulation weight, and *Resize* denotes broadcasting or expanding the channel vector along the spatial dimensions to match the spatial size of the input feature. This branch provides holistic scene-level semantic information to complement the local and multi-scale features, helping alleviate response confusion caused by complex textured backgrounds.

In summary, the three branches of HCAM model the input feature from the perspectives of fine-grained structure, multi-scale context, and global semantics, respectively. The Local Branch preserves spatially detailed responses, the Scale-aware Branch coordinates information from multiple receptive fields at different spatial locations, and the Global Branch provides global semantic cues. By concatenating and performing weighted fusion of these three complementary types of features, HCAM obtains a more comprehensive and hierarchical representation of defect features.

## 4. Experimental result and discussion

### 4.1. Experimental setting

#### (1) Dataset.

To evaluate the performance and cross-dataset applicability of the proposed method in few-shot industrial defect segmentation, experiments were conducted on two representative benchmarks, namely FSSD-12 and Surface Defects-4i.

The FSSD-12 dataset comprises images from 12 common categories of industrial surface defects [25]. To evaluate the model’s generalization to unseen defect categories, the dataset is divided into three mutually exclusive folds, with each fold containing four defect categories. During evaluation, the categories in one-fold are treated as unseen test categories, while those in the remaining folds are used for training.

Surface Defects-4i contains surface-defect images collected from multiple industrial materials, including aluminum, magnetic tiles, steel, leather, rails, and ceramic tiles [40]. Similar to FSSD-12, it is divided into three non-overlapping folds, each containing four defect categories, following the official class-wise evaluation protocol.

For consistent training and evaluation, all input images and annotation masks from both datasets are processed at a resolution of 200×200 pixels. The detailed category splits of the two datasets are summarized in Table 1.

**Table 1:** Dataset split details for FSSD-12 and Surface Defects-4i.

Fold	FSSD-12 defect categories	Surface Defects-4i defect categories
Fold-0	Am, Ia, Ld, Op	Al_Rm, MT_Uneven, Steel_Ld, Steel_Am
Fold-1	Os, Ws, Pa, Pk	Leather, Steel_Pa, Rail, Steel_Sc
Fold-2	Ri, Rp, Sc, Se	Al_Con, MT_Break, MT-Fray, tile

This class-wise partition ensures that the training and test categories do not overlap, allowing the model’s ability to segment previously unseen defect categories to be evaluated under both the 1-shot and 5-shot settings.

#### (2) Evaluation metrics.

To conduct a comprehensive and effective evaluation of the proposed method, two representative performance metrics commonly employed in the field of few-shot segmentation are adopted, namely the mean Intersection over Union (mIoU) and the Foreground-Background IoU (FB-IoU) [23]. The mIoU represents the arithmetic mean of the IoU scores across all defect categories, providing an overall measure of the model’s segmentation accuracy in multi-class defect scenarios. Its computation is formulated as follows:


$$\begin{array}{c} \mathrm{mIoU}=\frac{1}{C}\sum_{i=1}^{C}\frac{{TP}_{i}}{{TP}_{i}+{FP}_{i}+{FN}_{i}} \end{array}$$ (29)

where *C* denotes the total number of defect categories. For the *i*-th category, *TP*_*i*_ represents the number of pixels correctly predicted as belonging to that category, *FP*_*i*_ represents the number of pixels incorrectly predicted as that category but actually belonging to others, and *FN*_*i*_ represents the number of pixels actually belonging to that category but predicted as others. The FB-IoU metric evaluates the average performance of the model in distinguishing foreground (defect regions) from background (non-defect regions), thereby effectively reflecting the model’s discriminative capacity between target areas and their surrounding context. Its computation is formulated as follows:


$$\begin{array}{c} FB\text{-}IoU=\frac{{IoU}_{fg}+{IoU}_{bg}}{2} \end{array}$$ (30)

where *IoU*_*fg*_ denotes the IoU of the foreground defect regions, and *IoU*_*bg*_ represents the IoU of the background non-defect regions. By jointly considering the two aforementioned metrics, the performance of the proposed method in few-shot industrial defect segmentation scenarios can be comprehensively and objectively evaluated from two complementary perspectives: multi-class segmentation accuracy and foreground-background discrimination capability.

#### (3) Implementation details.

To ensure the fairness and reproducibility of the experiments, all comparative evaluations were conducted on a unified platform with the detailed configuration presented as follows. Input images were uniformly resized to 200 × 200 pixels during both training and inference. Models were trained for 200 epochs using the SGD optimizer with a momentum of 0.9. The initial learning rate was set to 0.001 and updated following an exponential decay schedule with a decay factor of 0.9 and a lower bound of $1\times 10^{-6}$. For conventional trainable CNN-based methods, ResNet-50 initialized with ImageNet-pretrained weights was used as the backbone, and the models were trained for 200 epochs. For Transformer- and SAM-based methods, their official architectures and pretrained settings were retained. To avoid interference from backbone parameter updates and to maintain feature consistency, the backbone parameters remained frozen throughout the entire training process. The experiments were conducted on an NVIDIA GeForce GTX Titan with 24 GB of memory, running the Ubuntu 20.04 LTS operating system. All models were implemented based on the PyTorch framework. The detailed parameter specifications are summarized in the Table 2.

**Table 2:** Experimental Setup.

Hardware	GPU	GeForce GTX Titan
Software	Ubuntu	20.04 LTS
Training strategy	Epoch	200
	Optimization algorithm	SGD
	momentum	0.9
	Learning rate	0.001
	Backbone	ResNet-50

### 4.2. Comparison with other methods

To validate the superiority of the proposed method, thirteen representative state-of-the-art few-shot segmentation models were selected for comparison, including CANet [24], PGNet [34], PMMs [38], PFENet [35], SCL [39], DCP [41], SCCAN [42], CPANet [25], TGRNet [40], BGNet [43], MAPTNet [44], Matcher [45], and PerSAM [46]. For the earlier methods, namely CANet, PGNet, and PMMs, previously published benchmark results were adopted. The remaining methods were evaluated using consistent dataset splits, shot settings, input resolutions, and evaluation protocols. For conventional trainable models, the training strategies were kept consistent where applicable, whereas the official architectures and pretrained settings were retained for Transformer- and SAM-based methods. Based on these experimental settings, a comprehensive quantitative and qualitative performance comparison was conducted, as detailed below.

#### (1) Evaluation on FSSD-12 dataset.

To quantitatively evaluate the performance advantages of the proposed method, the mIoU and FB-IoU metrics of all compared models on the FSSD-12 dataset are presented and compared. The experimental results are summarized in Table 3, where the best value in each column is highlighted in bold for intuitive comparison.

**Table 3:** Quantitative Comparison of mIoU and FB-IoU on the FSSD-12 Dataset.

Method	mIoU (1-shot)				FB-IoU (1-shot)	mIoU (5-shot)				FB-IoU (5-shot)
	Fold-0	Fold-1	Fold-2	Mean		Fold-0	Fold-1	Fold-2	Mean	
CANet_2019_	54.4	54.2	48.2	52.3	67.8	56.1	56.0	51.2	54.5	69.2
PGNet_2020_	57.9	46.4	52.3	52.2	67.5	49.3	54.2	53.9	52.5	70.1
PMMs_2020_	56.7	52.7	40.9	50.1	66.7	56.9	52.9	41.2	50.4	67.2
PFENet_2020_	51.0	34.4	37.3	40.9	61.3	51.8	34.3	38.0	41.4	61.4
SCL_2021_	58.8	53.5	47.3	53.2	70.7	57.8	52.4	47.9	52.7	70.3
DCP_2022_	50.1	25.5	33.8	36.5	60.6	50.6	25.3	35.8	37.2	60.9
SCCAN_2023_	55.6	58.5	54.3	56.1	68.2	54.8	59.2	54.4	56.1	68.7
CPANet_2023_	59.2	54.6	53.2	55.7	71.7	52.3	53.5	50.5	52.1	68.4
MAPTNet_2025_	68.2	58.2	55.3	60.6	75.6	63.6	**62.5**	52.9	59.7	73.5
Matcher_2024_	34.7	47.6	21.4	34.6	34.6	38.8	54.3	25.8	39.6	55.8
PerSAM_2024_	44.6	36.0	39.7	40.1	48.1	54.2	36.0	39.6	43.3	52.2
FH-Net	**70.8**	**59.2**	**58.0**	**62.7**	**76.1**	**71.1**	59.8	**60.0**	**63.6**	**76.6**

As shown in Table 3, FH-Net achieves the highest three-fold mean mIoU and FB-IoU under both the 1-shot and 5-shot settings. In the 1-shot setting, FH-Net obtains a mean mIoU of 62.7% and an FB-IoU of 76.1%, outperforming the strongest competing method, MAPTNet, by 2.1 and 0.5 percentage points, respectively. In the 5-shot setting, FH-Net achieves a mean mIoU of 63.6% and an FB-IoU of 76.6%, exceeding MAPTNet by 3.9 and 3.1 percentage points. Compared with its 1-shot results, the 5-shot performance improves by 0.9 percentage points in mIoU and 0.5 percentage points in FB-IoU, indicating that FH-Net can effectively utilize additional support samples. At the individual-fold level, FH-Net ranks first across all three folds under the 1-shot setting. Under the 5-shot setting, it achieves the best results on Fold-0 and Fold-2, while MAPTNet performs better on Fold-1 by 2.7 percentage points. Although FH-Net does not lead on every individual split, it maintains the highest three-fold mean performance under both shot settings, demonstrating balanced performance across different defect-category partitions. Moreover, its advantages over the Transformer-based MAPTNet and the SAM-based Matcher and PerSAM show that FH-Net remains competitive against recent Transformer and foundation-model approaches. Overall, these results verify the effectiveness and competitiveness of FH-Net for few-shot defect segmentation on FSSD-12.

**Fig 5:**
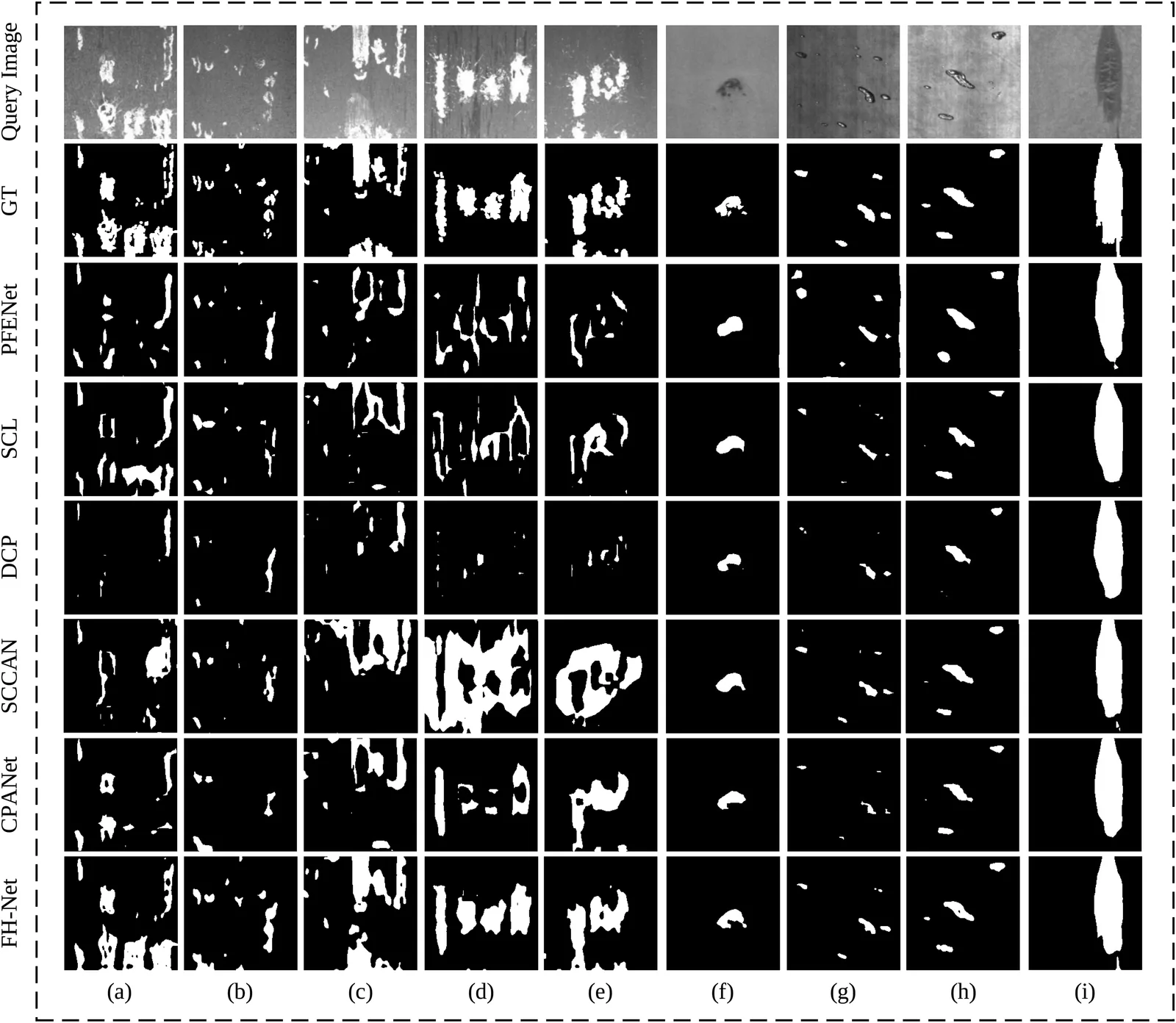
Qualitative comparison of different methods on the FSSD-12 dataset under the 1-shot setting. White and black pixels denote defect and background regions, respectively.

Fig 5 presents a qualitative comparison of FH-Net with five representative competing methods: PFENet, SCL, DCP, SCCAN, and CPANet. The figure shows the defect segmentation results of different methods under the 1-shot setting, enabling an intuitive comparison in terms of defect recognition, defect-region completeness, and boundary localization. As shown in Fig 5, FH-Net generally produces more accurate and complete defect predictions than the compared methods. Specifically, for the large-area defect regions shown in columns (a), (c), (d), (e), and (i), the segmentation results produced by FH-Net align more closely with the ground-truth annotations and exhibit clearer boundary delineation. Compared with PFENet, SCL, DCP, and CPANet, FH-Net yields more complete defect regions. In comparison with SCCAN, FH-Net provides more accurate segmentation without noticeable over-segmentation artifacts. For the small-sized defects presented in columns (b), (g), and (h), FH-Net demonstrates fewer false positives and missed regions relative to the other models. These observations are consistent with the intended roles of SPFP and HCAM. HCAM integrates contextual information across different levels and scales, strengthening the representation of defects with substantial scale variations. Meanwhile, SPFP enriches foreground-related support representations through frequency-domain perturbation, helping the model accommodate defects with diverse morphologies while preserving their structural information. In summary, the qualitative results show that FH-Net provides more complete defect-region recovery and more accurate boundary localization, offering intuitive evidence of its effectiveness and competitiveness in few-shot industrial defect segmentation.

#### (2) Evaluation on Surface Defects-4i dataset.

To further evaluate the cross-dataset applicability of FH-Net, its quantitative performance was compared with representative few-shot segmentation methods on the Surface Defects-4i dataset under both the 1-shot and 5-shot settings. The results are presented in Table 4, where the best result in each column is highlighted in bold.

**Table 4:** Quantitative Comparison of mIoU and FB-IoU on the Surface Defects-4i Dataset.

Method	mIoU (1-shot)				FB-IoU (1-shot)	mIoU (5-shot)				FB-IoU (5-shot)
	Fold-0	Fold-1	Fold-2	Mean		Fold-0	Fold-1	Fold-2	Mean	
CANet_2019_	25.3	20.1	12.6	19.3	49.4	27.1	22.4	14.3	21.3	53.1
PGNet_2020_	20.0	19.4	21.3	20.3	48.9	22.3	20.0	21.6	21.3	50.0
PMMs_2020_	30.8	19.6	11.5	20.6	49.0	30.5	20.8	19.7	23.6	51.3
PFENet_2020_	31.2	25.1	20.5	25.6	46.4	32.8	29.8	23.9	28.8	51.3
SCL_2021_	31.9	29.6	22.3	27.9	44.0	33.1	32.2	23.8	29.7	46.6
DCP_2022_	31.4	26.1	20.3	25.9	48.6	31.6	27.4	19.4	26.1	49.4
CPANet_2023_	37.3	35.0	21.3	31.2	53.6	26.8	34.0	**26.3**	29.0	54.5
TGRNet_2021_	40.9	35.6	21.6	32.7	50.8	37.3	30.2	25.6	31.0	50.8
BGNet_2025_	**46.0**	28.6	**24.6**	33.1	50.5	41.6	26.9	25.0	31.2	47.9
MAPTNet_2025_	42.0	41.8	21.6	35.1	54.2	48.4	45.4	23.7	**39.2**	57.1
Matcher_2024_	44.5	23.3	17.8	28.5	51.6	**52.0**	24.5	24.9	33.8	52.3
FH-Net	42.0	**45.4**	20.0	**35.8**	**56.6**	47.2	**47.7**	21.7	38.9	**59.4**

As shown in Table 4, FH-Net achieves the highest mean mIoU and FB-IoU under the 1-shot setting, reaching 35.8% and 56.6%, respectively. Compared with the strongest competing method, MAPTNet, FH-Net improves these two metrics by 0.7 and 2.4 percentage points. Although FH-Net does not achieve the best mIoU on every individual fold, it obtains the best result on Fold-1 and the highest three-fold mean performance, demonstrating competitive segmentation performance across different defect-category partitions. Under the 5-shot setting, FH-Net achieves a mean mIoU of 38.9%, ranking second and trailing MAPTNet by only 0.3 percentage points. Nevertheless, FH-Net obtains the highest FB-IoU of 59.4%, exceeding MAPTNet by 2.3 percentage points. Compared with its 1-shot results, its mean mIoU and FB-IoU increase by 3.1 and 2.8 percentage points, respectively, indicating that the model benefits from additional support information. FH-Net also maintains clear overall advantages over the SAM-based Matcher under both shot settings. Overall, despite performance variations across individual folds, FH-Net achieves the best or competitive mean results under both shot settings, supporting the cross-dataset applicability of the complete architecture on Surface Defects-4i.

**Fig 6:**
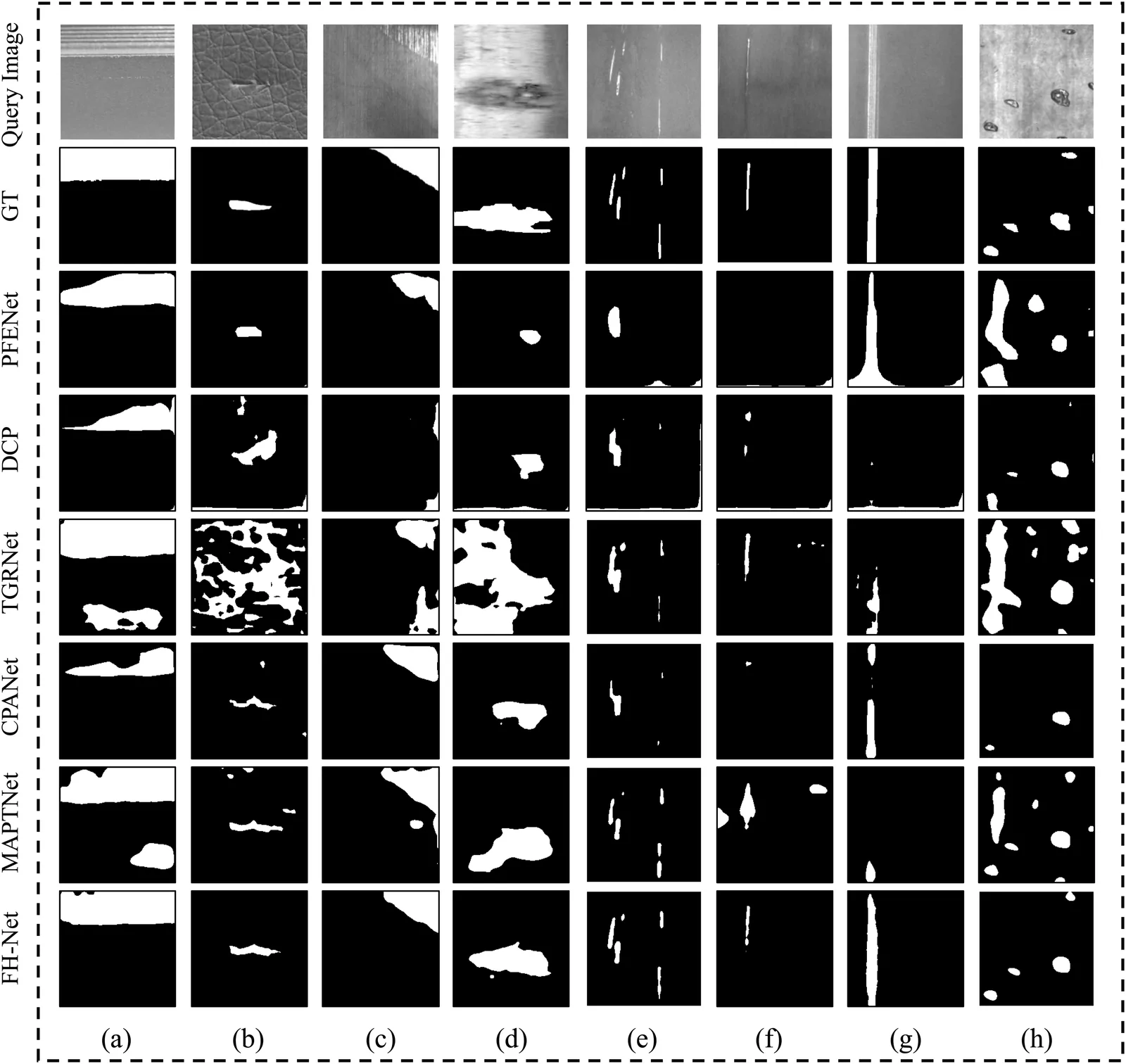
Qualitative comparison of different methods on the Surface Defects-4i dataset under the 1-shot setting. White and black pixels denote defect and background regions, respectively.

Fig 6 presents a qualitative comparison of FH-Net with five representative competing methods on the Surface Defects-4i dataset: PFENet, DCP, TGRNet, CPANet, and MAPTNet. The figure shows the segmentation results under the 1-shot setting, enabling an intuitive comparison in terms of defect-region completeness, boundary localization, and background interference. As shown in Fig 6, FH-Net generally produces more accurate and complete predictions for defects with different areas and morphologies. For the large-area or boundary-connected defects shown in columns (a) and (c), FH-Net accurately recovers the principal defect regions without introducing noticeable background false positives. In columns (b) and (d), its predictions more closely match the locations and overall shapes of the ground-truth annotations, whereas TGRNet exhibits substantial over-segmentation and PFENet and DCP recover only part of the defect regions. For the slender or small-area defects in columns (e)–(g), FH-Net better preserves the continuity and morphology of the defect structures, with fewer missed or excessively expanded regions. In column (h), FH-Net recovers multiple disconnected defect regions while effectively suppressing background false positives.

Together with the results on FSSD-12, these qualitative findings show that FH-Net maintains competitive segmentation performance across industrial defect datasets with different defect morphologies and background characteristics, providing further evidence of its robustness and generalizability under few-shot settings.

#### (3) Model accuracy and complexity.

To further evaluate the feasibility of the proposed method for practical deployment, Table 5 presents a comprehensive comparison between FH-Net and several representative few-shot segmentation models from five perspectives: number of parameters, computational complexity, inference latency, throughput, and segmentation accuracy. All metrics are measured on the Fold-0 subset of the FSSD-12 dataset under the 1-shot setting.

**Table 5:** Comparison of model complexity and accuracy.

Method	Parameters(M)	FLOPs(G)	Inference(ms)	FPS	Fold-0 mIoU (%)
PFENet_2020_	34.4	36.7	14.2	70.6	51.0
SCL_2021_	35.5	39.7	17.4	57.4	58.8
DCP_2022_	35.0	49.6	28.4	35.1	50.1
SCCAN_2023_	34.9	48.2	29.4	34.0	55.6
CPANet_2023_	30.3	74.2	19.2	52.2	59.2
FH-Net	45.4	73.1	27.9	35.8	70.8

From the perspective of model efficiency, PFENet, benefiting from its lightweight design, achieves the highest inference speed of 70.6 FPS with 34.4M parameters and 36.7G FLOPs. However, its mIoU reaches only 51.0%, indicating insufficient segmentation accuracy. SCL attains a relatively balanced trade-off between efficiency and accuracy, delivering 57.4 FPS with 35.5M parameters and an mIoU of 58.8%. CPANet possesses the smallest parameter count of 30.3M but incurs the highest FLOPs of 74.2G, yielding an inference speed of 52.2 FPS and an mIoU of 59.2%. Both DCP and SCCAN exhibit inference speeds below 36 FPS, reflecting weaker real-time capability. The proposed FH-Net contains 45.4M parameters, requires 73.1G FLOPs, and operates at an inference speed of 35.8 FPS. Compared with the aforementioned lightweight models, FH-Net incurs moderately higher computational overhead; however, it achieves a segmentation accuracy of 70.8% mIoU, surpassing the second-best model CPANet by 11.6% and attaining the optimal performance among all compared methods. Its moderate inference speed remains sufficient to meet the basic real-time requirements of industrial online inspection.

In summary, FH-Net trades a reasonable increase in computational cost for a substantial gain in segmentation accuracy, striking a favorable balance between model complexity and inspection precision. It achieves the best overall performance among all evaluated methods.

### 4.3. Ablation study

#### (1) Effectiveness of each module.

To further validate the effectiveness of the proposed SPFP and HCAM modules, a series of ablation experiments were conducted on the FSSD-12 dataset. All experiments were performed under the 1-shot setting with ResNet-50 uniformly adopted as the backbone network. Quantitative and qualitative analyses were carried out to assess the contribution of each module to the overall performance, with the configuration excluding both modules serving as the baseline.

The experimental results are summarized in Table 6. As shown in the table, both the individual and joint incorporation of SPFP and HCAM lead to improvements in mIoU and FB-IoU over the baseline. Specifically, when SPFP is added alone, the mIoU values across the three folds reach 62.6%, 56.1%, and 54.9%, yielding a mean mIoU of 57.9%, which corresponds to a 3.4% improvement over the baseline. The mean FB-IoU attains 70.6%, a 3.2% gain relative to the baseline. These results confirm the effectiveness of SPFP, demonstrating that it enriches feature representations and enhances model learning capacity while mitigating the performance degradation induced by overfitting. With the sole addition of HCAM, the mean mIoU is 57.5% and the mean FB-IoU is 72.2%, reflecting improvements of 3.0% and 4.8% over the baseline, respectively. Consistently superior results are also observed across individual folds. This enhancement is attributed to the multi-granularity feature extraction branches of HCAM, which strengthen the representation of complex features, and to the contribution-based weighted fusion that further enriches the feature information, ultimately boosting segmentation performance. When SPFP and HCAM are jointly incorporated, the model achieves optimal performance. Compared with the baseline, the mIoU increases by 12.0%, 5.2%, and 7.4% across the three folds, with a mean gain of 8.2%. The FB-IoU improves by 11.6%, 9.6%, and 4.7% on the respective folds, yielding a mean gain of 8.7%. These findings indicate that SPFP and HCAM not only contribute significantly when applied individually but also exhibit favorable synergistic effects when combined.

**Table 6:** Ablation study results on the FSSD-12 dataset.

SPFP	HCAM	mIoU (1-shot)				FB-IoU (1-shot)			
		Fold-0	Fold-1	Fold-2	Mean	Fold-0	Fold-1	Fold-2	Mean
**–**	**–**	58.8	54.0	50.6	54.5	72.3	58.3	71.7	67.4
✓	**–**	62.6	56.1	54.9	57.9	78.6	59.1	74.0	70.6
**–**	✓	63.4	54.3	54.7	57.5	79.1	61.8	75.7	72.2
✓	✓	70.8	59.2	58.0	62.7	83.9	67.9	76.4	76.1

**Fig 7:**
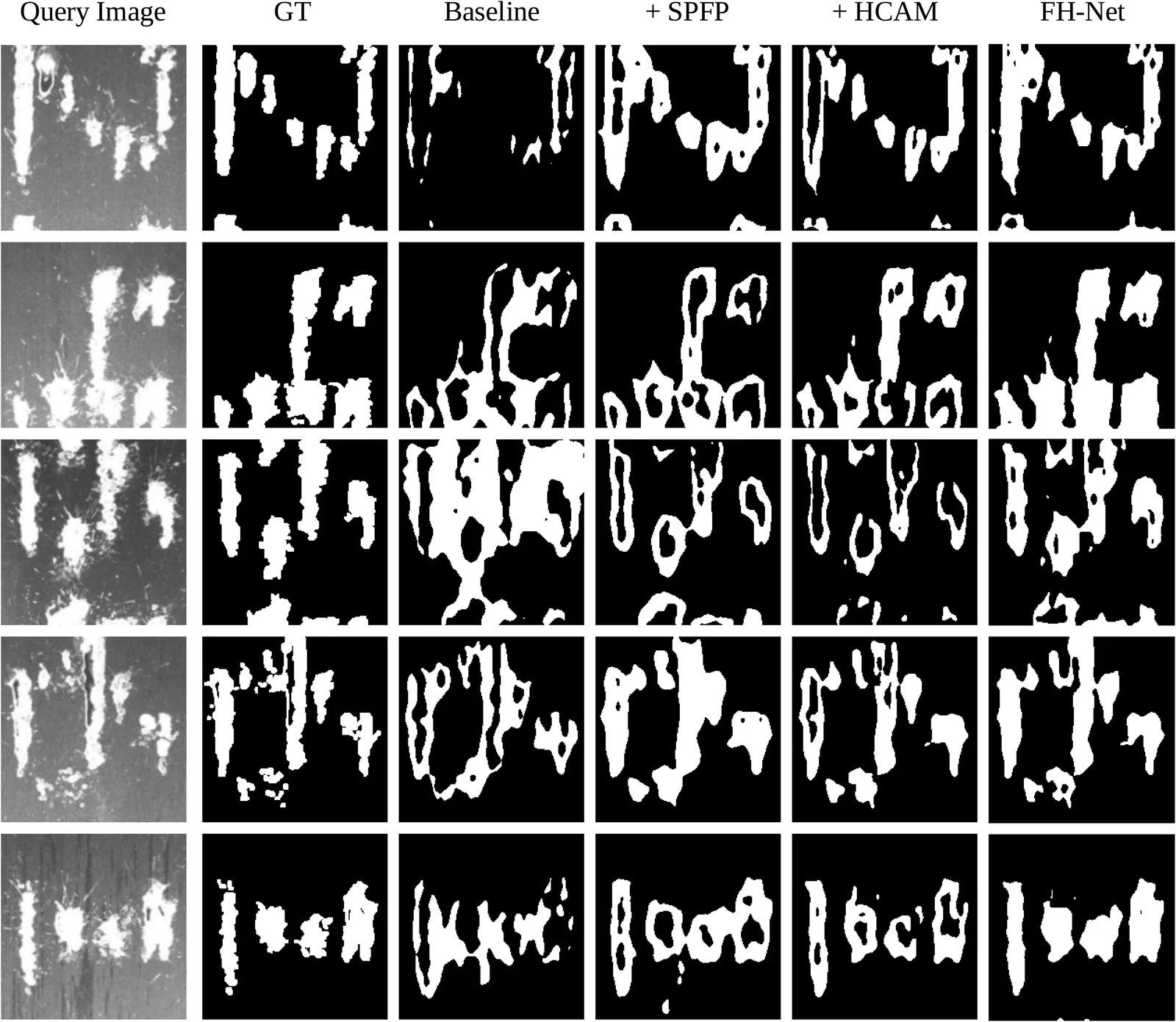
Ablation study results on the FSSD-12 dataset.

To further investigate the influence of different modules on the overall segmentation performance, the segmentation outputs of the baseline model, the models with either SPFP or HCAM added individually, and the full FH-Net model incorporating both modules are visualized and compared for qualitative analysis. The segmentation results are illustrated in Fig 7. As can be observed, the baseline model exhibits varying degrees of mis-segmentation compared with the ground truth annotations. Specifically, the baseline model frequently misclassifies large background regions outside the defect areas as defects, as shown in the third and fifth rows of the figure. Meanwhile, within the defect interiors, the baseline model often produces hollow or missing regions, as evident in the second, fourth, and sixth rows. These observations indicate that the baseline model possesses limited discriminative capacity for defects, insufficient extraction of large-scale features, and inadequate perception of small-scale details.

After the individual addition of either SPFP or HCAM, the segmentation performance is notably enhanced. On the one hand, the model exhibits improved discrimination against background regions outside the defects, resulting in reduced mis-segmented areas and sharper boundary delineation, as illustrated in the third and fifth rows. On the other hand, the coverage of defect interiors becomes more complete, with substantially diminished hollow areas and significantly increased recall of true defect regions, as demonstrated in the first, second, and fourth rows. This improvement suggests that the incorporation of a single module enables the model to extract and integrate multi-granularity feature information, thereby strengthening its capacity to learn and discriminate defects. When both SPFP and HCAM are jointly integrated, the defect regions segmented by FH-Net exhibit closer correspondence to the ground truth annotations in both morphology and location. This outcome further corroborates the effectiveness and synergistic contribution of the two core modules.

#### (2) Influence of probability parameters in the SPFP module.

**Fig 8:**
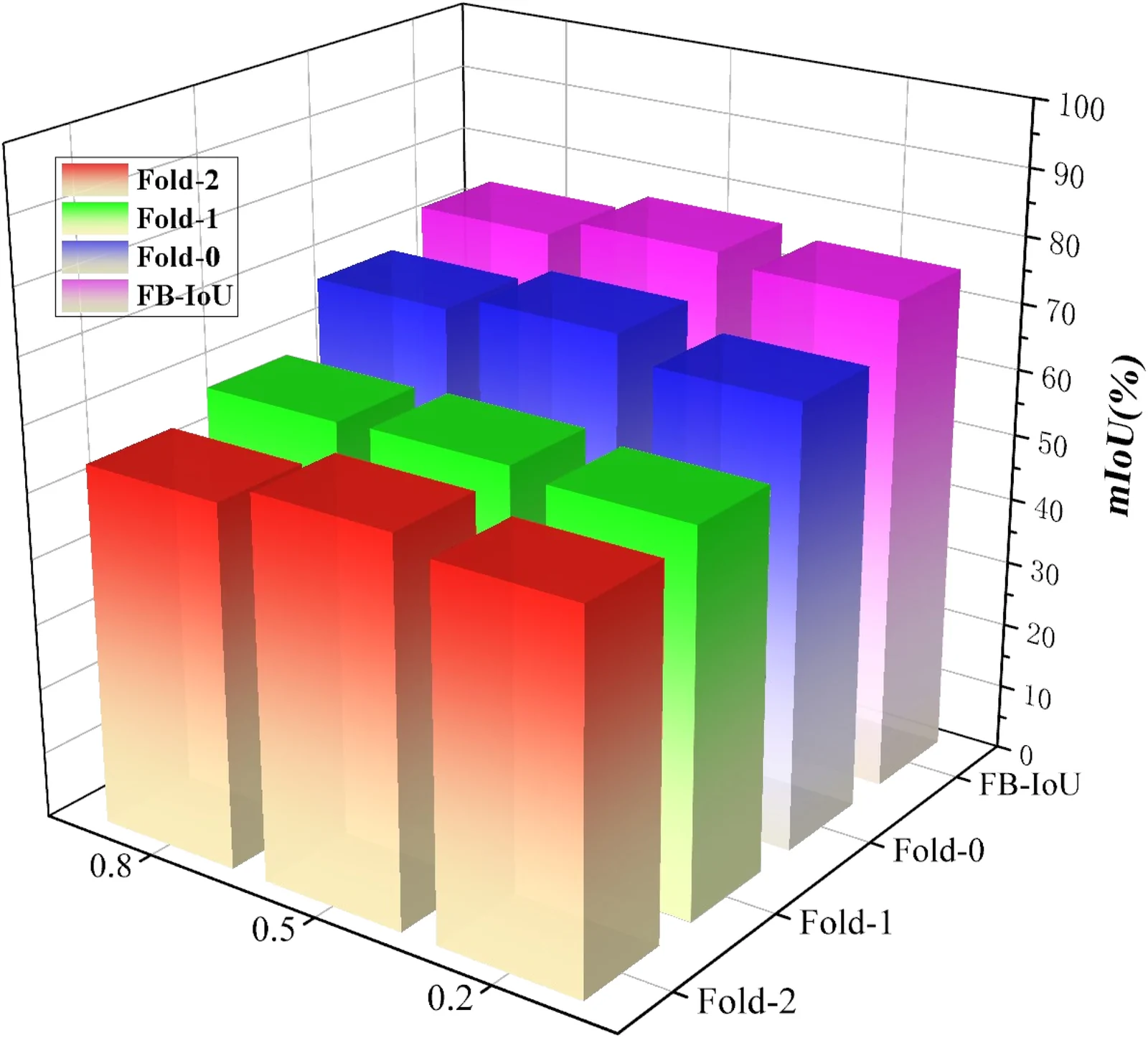
Ablation study results on the FSSD-12 dataset.

In the SPFP module, the probability parameter *prob* governs the proportion of features that undergo both intra-foreground and global-level frequency-domain perturbations. A smaller *prob* value restricts most features to intra-foreground perturbation only, whereas a larger value allows more features to receive dual-level perturbations. To systematically investigate the influence of this probability parameter on the overall segmentation performance and to determine its optimal setting, three ablation experiments were conducted within the FH-Net framework with *prob* set to 0.2, 0.5, and 0.8, respectively. The experimental results are presented in Table 7, and the corresponding bar chart derived from the experimental data is illustrated in Fig 8.

**Table 7:** Ablation study results of probability parameters.

Method	Parameter Value	mIoU (1-shot)			FB-IoU (1-shot)
		Fold-0	Fold-1	Fold-2	
+SPFP +HCAM	*prob* = 0.2	67.2	57.9	55.9	74.6
	*prob* = 0.5	70.8	59.2	58.0	76.1
	*prob* = 0.8	68.5	58.8	55.0	73.4

Comprehensive analysis of both the data table and the bar chart reveals that when *prob* is set to 0.5, the model achieves mIoU values of 70.8%, 59.2%, and 58.0% across the three folds, with a mean FB-IoU of 76.1%, consistently outperforming the settings of 0.2 and 0.8 and attaining the optimal segmentation performance. When the probability parameter is reduced to 0.2, the limited scope of global-level perturbation leads to insufficient feature diversity expansion, causing a performance decline. Conversely, when the parameter is increased to 0.8, excessive global perturbation may interfere with the original semantic structure, likewise resulting in degradation. These findings indicate that a moderate probability setting, with prob set to 0.5, strikes an effective balance between preserving fine-grained foreground details and introducing beneficial feature diversity, thereby maximizing the module’s contribution to model robustness and generalization capability.

#### (3) Comparison with alternative support-feature perturbation strategies.

Table 6 shows that incorporating SPFP alone improves the performance of the baseline model. However, it remains unclear whether this improvement arises from its task-adapted design or merely from the generic augmentation effect of feature perturbation. To address this issue, SPFP is compared with four alternative support-feature perturbation strategies. Feature Gaussian noise directly adds random noise to the support features. Fourier Domain Adaptation (FDA) [47] style perturbation modifies the low-frequency amplitude components. Fourier Augmented Co-Teacher (FACT) [48] style perturbation interpolates the entire amplitude spectrum. Mask-free Fourier perturbation removes the foreground constraint and applies frequency-domain perturbation to the complete support feature map. In these experiments, HCAM is disabled, and only the support-feature perturbation strategy is replaced, while the remaining network architecture, training configuration, and evaluation protocol are kept unchanged. The results are presented in Table 8.

**Table 8:** Comparison with different support-feature perturbation strategies on FSSD-12 under the 1-shot setting.

Perturbation strategy	mIoU (1-shot)				FB-IoU (1-shot)
	Fold-0	Fold-1	Fold-2	mean	
Gaussian noise	60.5	56.5	50.3	55.8	68.1
FDA	61.3	56.7	53.8	57.3	69.6
FACT	61.0	**56.8**	54.8	57.5	70.6
Mask-free Fourier	59.1	54.0	52.1	55.1	68.3
Full SPFP	**62.6**	56.1	**54.9**	**57.9**	**70.6**

The full SPFP achieves the highest mean mIoU of 57.9% and a mean FB-IoU of 70.6%, tying with FACT-style perturbation for the highest FB-IoU. Compared with feature Gaussian noise, SPFP improves the mean mIoU and FB-IoU by 2.1 and 2.5 percentage points, respectively. This result indicates that frequency-structured amplitude perturbation is more suitable for enhancing support features than unstructured random noise. By modifying the amplitude statistics of the support features while preserving their original phase information, SPFP may reduce the risk of disrupting the spatial structure of defects.

FDA-style and FACT-style perturbations achieve mean mIoU values of 57.3% and 57.5%, respectively, indicating that conventional Fourier amplitude manipulation can itself improve segmentation performance and may enhance support-feature diversity. SPFP improves upon these two strategies by 0.6 and 0.4 percentage points in mean mIoU, respectively. Its mean FB-IoU is 1.0 percentage point higher than that of FDA-style perturbation but equal to that of FACT-style perturbation. Therefore, the performance advantage of SPFP over the two Fourier-based baselines is modest and is not consistently observed across every fold and evaluation metric. The core amplitude operations of FDA and FACT were originally developed primarily for image-level domain adaptation and domain generalization, respectively. In contrast, SPFP applies foreground-guided frequency-domain perturbation to support features before prototype construction, thereby more directly enriching class-relevant support representations.

After the foreground constraint is removed, the mean mIoU and FB-IoU decrease to 55.1% and 68.3%, respectively, representing reductions of 2.8 and 2.3 percentage points relative to the full SPFP. A possible explanation is that indiscriminate perturbation introduces complex background variations into the subsequent prototype representation, whereas foreground guidance helps reduce such interference. These results suggest that the advantage of SPFP is associated with its foreground constraint and task-adapted perturbation of support features before prototype construction, rather than with the Fourier transform alone.

#### (4) Feature-statistical analysis of SPFP.

To further explain the feature changes associated with the performance gains of SPFP, amplitude-spectrum variation, foreground feature diversity, and phase consistency are statistically analyzed. HCAM is disabled during this experiment, and unique support samples from the three folds and their four feature levels are analyzed. Each feature observation is independently perturbed five times using SPFP. The identity reference denotes the comparison of an unchanged feature with itself; therefore, its relative amplitude-spectrum change and foreground feature diversity are both 0, whereas its phase consistency is 1. The results are reported as the mean ± standard deviation in Table 9.

**Table 9:** Feature-statistical effects of SPFP on FSSD-12 under the 1-shot setting.

Feature statistic	Identity reference	SPFP
Relative amplitude-spectrum change	0	0.71±0.30
Foreground feature diversity	0	0.37±0.12
Phase consistency	1	0.99±0.01

As shown in Table 9, the relative amplitude-spectrum change after SPFP reaches 0.71, indicating that SPFP substantially alters the amplitude statistics of the support features. Foreground feature diversity increases from 0 to 0.37, demonstrating that repeated perturbations expand the range of foreground representations derived from limited support samples. Its relatively large standard deviation also indicates that the degree of variation differs across samples and feature levels. Meanwhile, phase consistency remains at 0.99, indicating that SPFP largely preserves the original phase structure while introducing feature variations.

Overall, SPFP alters the amplitude statistics and increases foreground representation diversity while maintaining a stable phase structure. This finding is consistent with the segmentation performance improvements reported in Table 8 and further supports the effectiveness of its frequency-domain perturbation mechanism.

#### (5) Internal branch ablation and scale-weight visualization of HCAM.

Table 6 shows that incorporating HCAM alone improves the segmentation performance of the baseline model. However, the relationship between this improvement and the individual feature-extraction branches within HCAM remains unclear. Therefore, the Local Branch, Scale-aware Branch, and Global Branch are removed individually to evaluate the contributions of contextual information at different levels. In these experiments, SPFP is disabled, and only the HCAM configuration is changed, while all other network components, training configurations, and evaluation protocols are kept unchanged. The results are presented in Table 10.

**Table 10:** Ablation results for the internal branches of HCAM on FSSD-12 under the 1-shot setting.

HCAM configuration	mIoU (1-shot)				FB-IoU (1-shot)
	Fold-0	Fold-1	Fold-2	mean	
w/o Global Branch	60.2	**54.7**	53.2	56.0	71.6
w/o Local Branch	62.1	54.5	52.6	56.4	**72.7**
w/o Scale-aware Branch	60.0	52.5	54.1	55.5	70.9
Full HCAM	**63.4**	54.3	**54.7**	**57.5**	72.2

As shown in Table 10, the full HCAM achieves the highest mean mIoU of 57.5%. Removing the Global Branch reduces the mean mIoU and FB-IoU to 56.0% and 71.6%, corresponding to decreases of 1.5 and 0.6 percentage points, respectively. This result suggests that global semantic information provides holistic contextual cues that complement the local and multi-scale features and may help alleviate background confusion caused by relying solely on local responses. Removing the Local Branch decreases the mean mIoU by 1.1 percentage points to 56.4%. In particular, the mIoU values on Fold-0 and Fold-2 decrease by 1.3 and 2.1 percentage points, respectively, indicating that fine-grained local information contributes to preserving defect structures and detailed responses. However, the mean FB-IoU increases slightly from 72.2% to 72.7%, indicating that the contribution of the Local Branch is not consistent across all evaluation metrics and is mainly reflected in the improvement of the mean mIoU. The most pronounced performance degradation occurs when the Scale-aware Branch is removed. The mean mIoU and FB-IoU decrease to 55.5% and 70.9%, respectively, representing reductions of 2.0 and 1.3 percentage points. Moreover, the mIoU decreases across all three folds. These results demonstrate that the Scale-aware Branch plays an important role in handling variations in defect morphology and scale, as its multi-scale feature extraction and weighting mechanism enables the model to utilize contextual information from different receptive fields.

**Fig 9:**
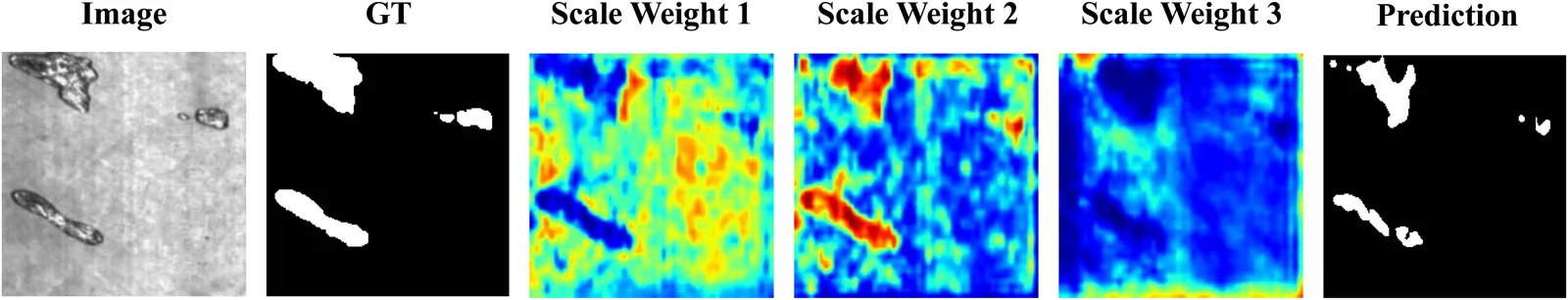
Visualization of the spatially varying scale weights generated by the Scale-aware Branch.

To further illustrate how the Scale-aware Branch coordinates information from different scales, Fig 9 visualizes the three spatial scale-fusion weight maps assigned to its scale-specific pathways, together with the final prediction. Warmer colors indicate larger relative contributions of the corresponding scale features at each spatial location. These maps represent scale-fusion weights rather than foreground probabilities.

As shown in Fig 9, the three scale-weight maps exhibit distinct spatial distributions. Scale Weight 2 assigns larger relative weights to several major defect regions, whereas Scale Weight 1 receives lower weights at the corresponding locations. At other spatial positions, the relative contributions of Scale Weights 1 and 3 increase, indicating that the corresponding scale-specific pathways provide complementary information. These observations indicate that the Scale-aware Branch does not combine the three scale-specific features using fixed or equal weights. Instead, it adaptively adjusts their relative contributions according to the features at different spatial locations. The final prediction recovers the major defect regions with different areas and morphologies, further illustrating the role of selective multi-receptive-field aggregation in defect segmentation.

Overall, the Local, Scale-aware, and Global Branches enhance the feature representation from the perspectives of fine-grained structure, multi-scale context, and global semantics, respectively. Although their contributions vary across folds and evaluation metrics, the full HCAM achieves the highest three-fold mean mIoU. The scale-weight visualizations further show that the Scale-aware Branch selectively coordinates information from different receptive fields at different spatial locations. Together, these quantitative and qualitative results support the complementarity of the three branches and the effectiveness of their joint use in HCAM.

#### (6) Comparison with conventional context aggregation and attention modules.

To further investigate whether the performance gains of HCAM arise merely from generic multi-scale feature enhancement or attention-based recalibration, we compare it with Atrous Spatial Pyramid Pooling (ASPP) [49] and the Convolutional Block Attention Module (CBAM) [50] through controlled module replacement. With SPFP disabled, HCAM was replaced by ASPP or CBAM at the same feature-processing positions, while the input–output dimensions and all other network and experimental settings were kept unchanged. The results are presented in Table 11.

**Table 11:** Comparison of HCAM with conventional context aggregation and attention modules on FSSD-12 under the 1-shot setting.

Module	mIoU (1-shot)				FB-IoU (1-shot)
	Fold-0	Fold-1	Fold-2	mean	
CBAM	60.0	56.1	50.4	55.5	70.7
ASPP	63.0	**58.1**	50.1	57.1	70.7
HCAM	**63.4**	54.3	**54.7**	**57.5**	**72.2**

As shown in Table 11, HCAM achieves the highest mean mIoU and FB-IoU, reaching 57.5% and 72.2%, respectively. Compared with CBAM, HCAM improves these two metrics by 2.0 and 1.5 percentage points, respectively. Although CBAM outperforms HCAM by 1.8 percentage points on Fold-1, HCAM exceeds CBAM by 3.4 and 4.3 percentage points on Fold-0 and Fold-2, respectively. CBAM recalibrates existing feature responses through channel and spatial attention, but this generic enhancement mechanism may be insufficient to explicitly coordinate the local structures, scale variations, and global context of industrial defects. In contrast, HCAM extracts information at different levels through its three branches, producing a more complementary feature representation and achieving higher overall performance.

Compared with ASPP, HCAM improves the mean mIoU and FB-IoU by 0.4 and 1.5 percentage points, respectively. ASPP achieves a higher mIoU on Fold-1, whereas HCAM outperforms it by 0.4 and 4.6 percentage points on Fold-0 and Fold-2, respectively. ASPP primarily aggregates information from different receptive fields using parallel atrous convolutions with predefined dilation rates. In contrast, HCAM explicitly coordinates fine-grained structures, multi-scale context, and global semantics through its Local, Scale-aware, and Global Branches. Therefore, the advantage of HCAM does not arise solely from receptive-field expansion, but from the coordinated use of contextual information at different levels, enabling it to achieve higher three-fold mean mIoU and FB-IoU.

Overall, HCAM achieves the highest three-fold mean mIoU and FB-IoU among the compared modules. Together with the results in Table 10, these findings support the effectiveness of jointly aggregating local, multi-scale, and global information beyond generic multi-scale enhancement or attention recalibration.

### 4.4. Performance analysis on extremely small defects

To further evaluate the segmentation performance of the proposed method on small defect regions, the area ratio of each connected defect region to the entire image is used as the size criterion. Three area-ratio thresholds, namely 1%, 3%, and 5%, are considered, and defect regions with area ratios below the corresponding thresholds are included in the evaluation. The 1% threshold represents the strictest criterion for extremely small defects, whereas the 3% and 5% thresholds are used to examine the stability of the results under different area criteria. All input images, ground-truth masks, and prediction maps have a resolution of 200×200 pixels. For each defect region satisfying the specified threshold, the overlapping predicted region with the highest IoU is selected, and the IoU between the two regions is calculated. If no predicted region overlaps with the defect region, its IoU is set to zero. Finally, the IoU values of all defect regions satisfying each threshold are averaged to obtain the mean small-defect region IoU. The experimental results are presented in Table 12.

**Table 12:** Comparison of mean small-defect region IoU(%) under different area thresholds on FSSD-12 in the 1-shot setting.

Defect-region area ratio threshold	Mean small-defect region IoU					
	DCP	PFENet	SCCAN	CPANet	SCL	FH-Net
< 1%	10.8	18.6	21.5	18.0	20.1	**31.4**
< 3%	16.4	23.2	25.2	25.1	25.5	**38.9**
< 5%	17.7	24.3	25.8	27.2	26.9	**41.0**

As shown in Table 12, FH-Net achieves the highest mean small-defect region IoU under all three area-ratio thresholds. When the defect-region area ratio is below 1%, FH-Net obtains an IoU of 31.4%, outperforming the best-performing competing method, SCCAN, by 9.9 percentage points. Under the 3% and 5% thresholds, FH-Net achieves IoU values of 38.9% and 41.0%, exceeding the corresponding best competing results by 13.4 and 13.8 percentage points, respectively.

As the area-ratio threshold decreases from 5% to 1%, the IoU values of all methods generally decline, indicating that smaller defect regions are more difficult to segment. Nevertheless, FH-Net maintains a clear performance advantage across all three thresholds, demonstrating its stronger ability to identify and segment small defect regions under limited-support conditions.

To provide an intuitive comparison of the ability of different methods to segment small defect regions, SCCAN, the best-performing competing method under the area-ratio threshold of 1%, is selected for qualitative comparison with FH-Net. The small defect regions marked by red boxes are further enlarged to facilitate the examination of differences in defect recognition, contour recovery, and boundary localization. It should be noted that these enlarged views are provided solely for visualization and are not used during model inference or quantitative evaluation.

**Fig 10:**
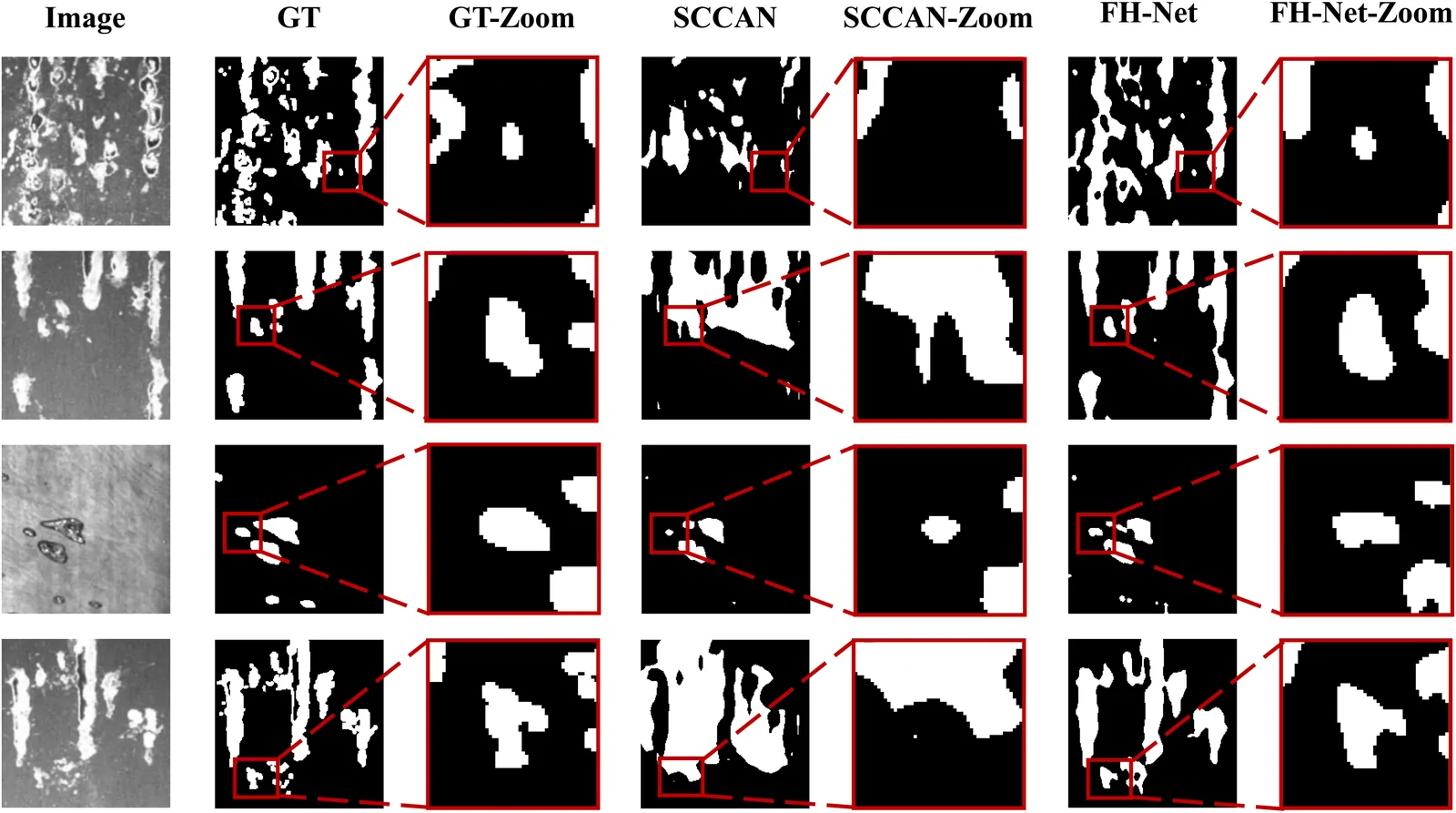
Qualitative comparison of small-defect region segmentation results on FSSD-12 under the 1-shot setting. The red boxes indicate defect regions occupying less than 1% of the image area, and the adjacent panels present the corresponding enlarged views.

As shown in Fig 10, in the first and fourth rows, SCCAN fails to correctly segment the small defects within the enlarged regions, whereas FH-Net successfully preserves the corresponding defect responses and produces predictions that more closely match the GT masks in both location and morphology. In the second and third rows, although SCCAN identifies the corresponding defect regions, its predictions exhibit noticeable over-segmentation or incomplete region coverage. In contrast, FH-Net produces more complete segmentations of the small defect regions, with contours and boundaries that are more consistent with the GT masks. These qualitative observations agree with the quantitative results in Table 12 and further demonstrate the advantage of FH-Net in recognizing and accurately segmenting small defects.

## 5. Conclusion

In the task of few-shot surface defect segmentation within industrial scenarios, existing methods confront two fundamental challenges. First, the extreme scarcity of support samples leads to insufficient feature diversity, rendering models prone to overfitting. Second, single-level feature extraction constrains the representational capacity for multi-scale and complex defects. To address these limitations, this paper presents a novel meta-learning-based segmentation framework termed FH-Net. By integrating a frequency-domain perturbation mechanism with a hierarchical feature aggregation strategy, FH-Net achieves high-precision segmentation of previously unseen defect categories using only a limited number of annotated samples. The main contributions are summarized as follows.

(1) A SPFP module is introduced. This module applies dual-level amplitude perturbations, at both the foreground and global scales, to support set features via Fourier transform. While strictly preserving the original semantic structure, SPFP enriches the diversity of prototype features, thereby enhancing the model’s generalization robustness and mitigating the risk of overfitting under few-shot conditions.
(2) A HCAM module is designed. It establishes a three-branch parallel architecture comprising local, scale-aware, and global pathways for multi-granularity feature extraction. An attention-driven adaptive weighting fusion mechanism is further incorporated, which strengthens the model’s discriminative representational capacity for multi-scale and irregular defects while reducing the loss of fine-grained spatial information.

Systematic experiments on two industrial few-shot surface defect segmentation benchmarks, FSSD-12 and Surface Defects-4i, validate the effectiveness and general applicability of FH-Net. On FSSD-12, FH-Net achieves three-fold mean mIoU values of 62.7% and 63.6% under the 1-shot and 5-shot settings, outperforming the corresponding second-best results by 2.1 and 3.9 percentage points, respectively. On Surface Defects-4i, FH-Net achieves a three-fold mean mIoU of 35.8% under the 1-shot setting, exceeding the second-best result by 0.7 percentage points. Under the 5-shot setting, it obtains a competitive mean mIoU of 38.9% and the highest FB-IoU of 59.4%, with the latter surpassing the second-best result by 2.3 percentage points. Ablation studies further quantify the substantial contributions of SPFP and HCAM, confirming their complementary and synergistic effects in enhancing feature richness and multi-scale representational capacity.

Nevertheless, FH-Net still exhibits limitations when handling extremely tiny defects or those heavily entangled with background textures. Future work may explore two promising directions. First, domain adaptation or domain generalization techniques could be introduced to improve the model’s transferability across diverse production lines and illumination conditions. Second, frequency-domain perturbation strategies may be combined with more efficient feature matching mechanisms to further heighten sensitivity to subtle defect signals.
